# A novel mouse model of creatine transporter deficiency

**DOI:** 10.12688/f1000research.5369.1

**Published:** 2014-09-29

**Authors:** Laura Baroncelli, Maria Grazia Alessandrì, Jonida Tola, Elena Putignano, Martina Migliore, Elena Amendola, Cornelius Gross, Vincenzo Leuzzi, Giovanni Cioni, Tommaso Pizzorusso

**Affiliations:** 1 Institute of Neuroscience, National Research Council (CNR), Pisa, I-56124, Italy; 2 Department of Developmental Neuroscience, IRCCS Stella Maris Scientific Institute, Calambrone (Pisa), I-56128, Italy; 3 Mouse Biology Unit, European Molecular Biology Laboratory (EMBL), Monterotondo (Roma), I-00015, Italy; 4 Department of Paediatrics, Child Neurology and Psychiatry, Sapienza University of Rome, Rome, I-00185, Italy; 5 Department of Clinical and Experimental Medicine, University of Pisa, Pisa, I-56126, Italy; 6 Department of Neuroscience, Psychology, Drug Research and Child Health NEUROFARBA, University of Florence, Florence, I-50135, Italy

## Abstract

Mutations in the creatine (Cr) transporter (CrT) gene lead to cerebral creatine deficiency syndrome-1 (CCDS1), an X-linked metabolic disorder characterized by cerebral Cr deficiency causing intellectual disability, seizures, movement  and behavioral disturbances, language and speech impairment ( OMIM #300352).

CCDS1 is still an untreatable pathology that can be very invalidating for patients and caregivers. Only two murine models of CCDS1, one of which is an ubiquitous knockout mouse, are currently available to study the possible mechanisms underlying the pathologic phenotype of CCDS1 and to develop therapeutic strategies. Given the importance of validating phenotypes and efficacy of promising treatments in more than one mouse model we have generated a new murine model of CCDS1 obtained by ubiquitous deletion of 5-7 exons in the *Slc6a8 *gene. We showed a remarkable Cr depletion in the murine brain tissues and cognitive defects, thus resembling the key features of human CCDS1. These results confirm that CCDS1 can be well modeled in mice. This CrT ^−/y^ murine model will provide a new tool for increasing the relevance of preclinical studies to the human disease.

## Introduction

The creatine (Cr) transporter (CrT, alias CRTR, MGC87396, CT1, SLC6A8, OMIM 300036) deficiency (CCDS1, OMIM #300352) is an X-linked inherited metabolic disorder characterized by cerebral Cr deficiency which results in intellectual disability, language and speech impairment, seizures and movement and behavioral disturbances, and affects about 1% of males with non- syndromic mental disability ( van de Kamp *et al.*, 2014). CrT loss of function is mostly caused by missense mutations and small deletions which are concentrated in the transmembrane domains 7 and 8 of the protein ( van de Kamp *et al.*, 2014). In physiological conditions, about half of our normal Cr requirement is satisfied by the diet. *De novo* endogenous synthesis of Cr takes place mainly in the kidney, liver and pancreas and involves the enzymes l-arginine: glycineamidinotransferase (AGAT) and S-adenosyl-l-methionine:N-guanidinoacetatemethyltransferase (GAMT) ( Wyss & Kaddurah-Daouk, 2000). Cr is a polar hydrophilic molecule unable to cross the lipidic membranes, which uses a Na ^+^- and Cl ^−^- dependent plasma membrane CrT to enter the cells ( Nash *et al.*, 1994). CrT is widely expressed in the brain tissue with a prominent presence in the cortical and subcortical regions involved in motor and sensory processing, learning and memory, and regulation of emotion-related behavior ( Lowe *et al.*, 2014; Mak *et al.*, 2009).

Patients affected by cerebral creatine deficiency syndrome-1 (CCDS1) share depletion of brain Cr and the clinical phenotype with patients carrying the other two defects of Cr metabolism which involve mutations of genes encoding the biosynthesizing enzymes AGAT and GAMT ( Item *et al.*, 2001; Stockler *et al.*, 1994). Replenishment of the brain Cr pool is the only effective therapy for Cr deficiency diseases ( Battini *et al.*, 2002; Schulze *et al.*, 2001; Stockler *et al.*, 1996). Unfortunately, in CCDS1 patients even very high doses of Cr, alone or combined with the Cr precursors arginine and glycine to stimulate endogenous Cr synthesis, fail to restore the Cr content in brain ( Chilosi *et al.*, 2008; Valayannopoulos *et al.*, 2012). There have been attempts to normalize the levels of Cr in the brain with Cr-lipophilic analogs, but these compounds have proven ineffective when administered to patients ( Fons *et al.*, 2010). Thus, CCDS1 is still missing an effective treatment.

Preclinical animal models are crucial tools to dissect disease pathogenic mechanisms and develop new therapeutic strategies. Only two murine models of CCDS1 are available so far, and they have only been analyzed at the behavioral and neurochemical level. An ubiquitous CrT knockout mouse model has been generated by deletion of 2–4 exons in the *Slc6a8* gene. Learning and memory deficits, impaired motor activity and Cr depletion in brain and muscles have been reported in animals at three-four months of age ( Skelton *et al.*, 2011). Another murine model is based on the use of the CaMKII promoter to drive Cre-recombinase expression, achieving a CrT deletion only in postnatal forebrain excitatory neurons. This strategy was successful in avoiding the peripheral Cr depletion and the motor deficits shown by germline CrT knockout mouse. Behavioral analysis in mice at 12 months of age revealed learning and memory impairments that could be ameliorated by supplementation of cyclocreatine, a Cr analog ( Kurosawa *et al.*, 2012).

For translational studies, the phenotype variations observed in different mouse models, carrying similar mutations and the effects of genetic backgrounds highlight the importance of validating phenotypes and therapeutic efficacy in multiple models and in different laboratories ( Katz *et al.*, 2012). Such validation will hopefully increase the relevance of preclinical studies to the human disease. To increase the number of CCDS1 models, we generated a novel murine model of CCDS1 obtained by ubiquitous deletion of 5–7 exons in the *Slc6a8* gene. These mice presented a remarkable Cr depletion in the brain tissue and displayed cognitive defects resembling the key features of human CCDS1, and providing a new promising CCDS1 animal model.

## Materials and methods

### Generation of CrT knockout mice

**Figure 1.**
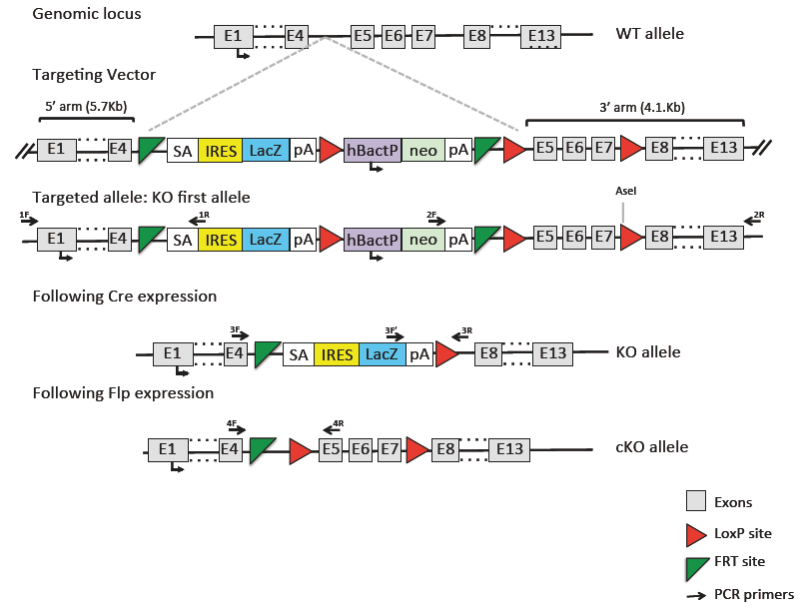
Sketch of the strategy for generation of a null Slc6a8 mouse. A targeting vector was obtained from KOMP to generate mice carrying a floxed allele. Crossing these mice with a Flp deleter mouse line produced a conditional KO mouse line (cKO allele). Crossing this line with a line expressing Cre-recombinase in the germline produced the Slc6a8 null mouse used in this study (KO allele). 1F, 1R, 2F, 2R, 3F, 3F’, 3R, 4F, 4R report the sites targeted by the PCR primers to assess allele presence.

A Cre-conditional allele of *Slc6a8* has been produced by introducing the loxP sites flanking exon 5–7 of the gene in embryonic stem (ES) cells via homologous recombination (vector PRPGS00081_A_A09 obtained from the NIH Knock-out Mouse Program, KOMP). The presence of lox sites has been checked by sequencing (sequencing service by MWG, Germany). The plasmid was linearized with NruI before electroporation into ES cells (129/Sv x C57BL/6N, clone A8, gift of A. Wutz, Wellcome Trust Centre for Stem Cell Research, Stem Cell Institute, University of Cambridge). G418-resistant clones were identified and screened by long-range PCR (Applied Biosystems Gene AMP PCR system 2700). Hybridization with a specific probe for the 5′ and 3′ arms was used to confirm the PCR results. Two independent positive ES cell clones were injected into C57BL/6N host embryos using a piezo-drill assisted 8-cell stage injection procedure developed at EMBL, Monterotondo Italy. Four out of five offspring (all >95% ES cell derived) provided germline transmission. Germline transmission of the allele was confirmed by long-range PCR and the neomycin selection cassette was removed by crossing with FLP recombinase expressing mice ( Farley *et al.*, 2000). Germline knockout mice were produced by crossing the constitutive allele to the HPLRT::Cre recombinase deleter mouse ( Tang *et al.*, 2002; Figure 1).

### Animal housing

Animals were maintained at 22°C under a 12-h light–dark cycle. Food and water were available *ad libitum*. All experiments were carried out in accordance with the European Communities Council Directive of 24 November 1986 (86/609/EEC) and were approved by the Italian Ministry of Health (authorization number 147/2014-B). All necessary efforts were made to minimize both stress and the number of animals used. As CrT deficiency is an X-linked pathology and only males are consistently affected, we focused our study on male animals. Young adult males (postnatal day P40 at the beginning of testing) of each genotype (CrT ^–/y^ mutants and CrT ^+/y^ wild-type littermates) were used in behavioral experiments, while a separate group of animals (P30) was assigned to Cr level assay.

### Detection of *Slc6a8* mutation by PCR

Genomic DNA was isolated from mouse tail using a kit, and the protocol suggested by the manufacturer (DNeasy Blood & Tissue Kit, Qiagen, USA). DNA was amplified for mutant and wild-type (WT) allele using a standard PCR protocol with the following primers: F:AGGTTTCCTCAGGTTATAGAGA; R:CCCTAGGTGTATCTAACATCT; R1: TCGTGGTATCGTTATGCGCC. For PCR amplification we used 300 ng of DNA in a 25 μL reaction volume containing 0.2 mM of each dNTP, 2 μM of F primer, 1 μM of R, 1 μM of R1 primer and 0.5 U/μL Red Taq DNA polymerase (Sigma-Aldrich, Italy). The PCR conditions were as follows: 94°C for 4 min followed by 37 cycles at 94°C for 30 s, 58°C for 30 s, 72°C for 40 s and a final extension at 72°C for 7 min. Amplicons were separated using 2% agarose gel and visualized under UV light after staining with Green Gel Plus (Fisher Molecular Biology, Rome, Italy). Amplicon sizes were: WT allele = 462 bp; mutant allele = 371 bp.

### Gas chromatography/mass spectrometry (GC/MS)

Mouse tissues, immediately frozen on dry ice and stored at -80°C until the analysis, were homogenized in 0.7 ml PBS buffer (Sigma-Aldrich, Italy) at 4°C using a ultrasonic disruptor (Microson Heat System, NY, USA) for brain or a glass manual homogenizer (VWR, Italy) for kidney, heart and muscle. After centrifugation (600 × g for 10 min at 4°C) an aliquot of the homogenate (50 µl) was assayed for protein content ( Lowry *et al.*, 1951), and the supernatant used for Cr assay as previously described ( Alessandrì *et al.*, 2005). Briefly, 50 µl of saturated sodium hydrogen carbonate and 50 µl of a mixture containing 2- phenylbutyric acid (I.S.) in toluene (6.09 mmol/l; Sigma-Aldrich, Italy) were added to 200 µl of homogenate. After adding 1 ml of toluene and 50 µl of hexafluoro-2,4-pentanedione (Sigma-Aldrich, Italy) to form bis-trifluoromethyl- pyrimidine derivatives, the mixture was stirred overnight at 80°C. The organic layer was centrifuged, dried under nitrogen and 2 µl of the residue derivatized at room temperature with 100 µl of BSTFA+TMCS (Sigma-Aldrich, Italy) injected into the GC/MS. GC analyses were performed using an Agilent 6890N GC equipped with an HP5MS capillary column (0.25 mm × 30 m, film thickness 0.25 ìm) and an Agilent mass spectrometer 5973N (Agilent Technologies, Italy). The mass spectrometer was set in EI- single ion monitoring mode (SIM). The ions with m/z of 192 for I.S., 258 for Cr and 225 for guanidinoacetic acid (GAA) were used for calculation of the metabolites, using standard curves ranging 5–90 µmol/L and 0.30–6 µmol/L for Cr and GAA, respectively. Data were processed by the G1701DA MSD ChemStation software. All the aqueous solutions were prepared using ultrapure water produced by a Millipore system.

### Behavioral testing

The testing order consisted of: open field (1 day duration), object recognition test (ORT) at 24h (3 days), Y maze (1 day), Morris water maze (MWM) with hidden platform (7 days), and locomotor activity (1 day). The mice were tested on one task at a time with the next behavioral test starting at least 2 days after the completion of the previous one. In order to reduce the circadian effects, all behavioral tests were performed during the same time interval each day (1400–1800h; light phase). All behavioral tests were conducted in blind with respect to the genotype of animals. Mice were weighed at the end of experiments (P60).

### Open field and object recognition test (ORT)

We followed the protocol reported in Lonetti *et al.*, 2010. Briefly, the apparatus consisted of a square arena (60 × 60 × 30 cm) constructed in poly(vinyl chloride) with black walls and a white floor. The mice received two sessions of 10-min duration in the empty arena on two consecutive days to habituate them to the apparatus and test room. Animal position was continuously recorded by a video tracking system (Noldus Ethovision XT). In the recording software an area corresponding to the center of the arena (a central square 30 × 30 cm), and a peripheral region (corresponding to the remaining portion of the arena) were defined. The total movement of the animal and the time spent in the center or in the periphery area were automatically computed. The mice activity during the first day of habituation was analyzed for evaluating the behavior in the open field arena. The ORT consisted of two phases: sample and testing phase. During the sample phase, two identical objects were placed in diagonally opposite corners of the arena, approximately 6 cm from the walls, and mice were allowed 10 min to explore the objects, then they were returned to their cage. The objects to be discriminated were made of plastic, metal, or glass material and were too heavy to be displaced by the mice. Arena and objects were cleaned with 10% ethanol between trials to stop the build-up of olfactory cues. The testing phase was performed 24h after the sample phase. One of the two familiar objects was replaced with a new one, while the other object was replaced by an identical copy. The objects were placed in the same locations as the previous ones. The mice were allowed to explore objects for 5 min. To avoid possible preferences for one of two objects, the choice of the new and old object and the position of the new one were randomized among animals. The amount of time spent exploring each object (nose sniffing and head orientation within <1.0 cm) was recorded and evaluated by the experimenter blind to the mouse genotype. Mice exploring the two objects for less than 10 s during the sample phase were excluded from testing. A discrimination index was computed as DI = (T _new_ - T _old_)/(T _new_ + T _old_), where T _new_ is the time spent exploring the new object, and T _old_ is the time spent exploring the old one.

### Y maze

Spontaneous alternation was measured using the Y-maze, as described in Begenisic *et al.*, 2014. We used a Y-shaped maze with three symmetrical grey solid plastic arms at a 120-degree angle (26 cm length, 10 cm width, and 15 cm height). Mice were placed in the center of the maze and allowed to freely explore the maze for 8 minutes. The apparatus was cleaned with 10% ethanol between trials to avoid the build-up of odor traces. All sessions were video-recorded for offline blind analysis. The arm entry was defined as all four limbs within the arm. A triad was defined as a set of three arm entries, when each entry was to a different arm of the maze. The number of arm entries and the number of triads were recorded in order to calculate the alternation percentage (generated by dividing the number of triads by the number of possible alternations and then multiplying by 100).

### Morris water maze

Mice were trained for four trials per day and for a total of 7 days in a circular water tank, made from grey polypropylene (diameter, 120 cm; height, 40 cm), filled to a depth of 25 cm with water (23°C) rendered opaque by the addition of a small amount of a non-toxic white paint. Four positions around the edge of the tank were arbitrarily designated North (N), South (S), East (E), and West (W), which provided four alternative start positions and also defined the division of the tank into four quadrants, i.e., NE, SE, SW, and NW. A square clear Perspex escape platform (11 × 11 cm) was submerged 0.5 cm below the water surface and placed at the midpoint of one of the four quadrants. The hidden platform remained in the same quadrant during training, while the start positions (N, S, E, or W) were randomized across trials. Mice were allowed up to 60 s to locate the escape platform, and their swimming paths were automatically recorded by the Noldus Ethovision system. On the last trial of the last training day, mice received a probe trial, during which the escape platform was removed from the tank and the swimming paths were recorded over 60 s while mice searched for the missing platform. The swimming paths were recorded and analyzed with the Noldus Ethovision system.

### Measurement of spontaneous locomotor activity

Opto M3 multi-channel activity monitors (Columbus Instruments, OH, USA) were used to quantify spontaneous horizontal activity of animals. Monitors were placed in the colony area and testing was conducted in the same conditions of animal facility housing. All measurements were performed from 6:00 P.M. to 6:00 A.M. (dark phase) and to 6:00 A.M. to 6:00 P.M. (light phase), using animals maintained on a 12 hr light/dark cycle from 6:00 A.M. to 6:00 P.M. Individual mice were placed in 33 × 15 × 13-cm (length × width × height) clear plastic cages for 24h and total distance travelled was calculated from infrared beam breaks by determining activity at 1-min intervals. Horizontal activity was measured by the sequential breaking of infrared beams, 2.54 cm on center, in the horizontal plane of the x axis.

### Statistical analysis

All statistical analyses were performed using SigmaStat Software. Differences between two groups were assessed with a two-tailed t test. The significance of factorial effects and differences among more than two groups were evaluated with ANOVA/RM ANOVA followed by Holm-Sidak test. Rank transformation was exploited for data not normally distributed. The level of significance was p < 0.05.

## Results

### *CrT* deletion leads to significant Cr reduction in brain and other tissues

In order to determine the effectiveness of our approach for targeting *CrT* gene, the Cr levels were measured by GC/MS in various tissues. We observed a significant reduction of Cr in the brain (both cerebral cortex and hippocampus; Two Way ANOVA on ranks, post hoc Holm-Sidak method, p < 0.01 and p < 0.001 respectively), muscle (p < 0.01), heart (p < 0.001) and kidney (p < 0.05) of CrT ^−/y^ mice with respect to wild-type (WT) littermates (n = 4/tissue for each group; Table 1). To ensure that kidney Cr reduction was not due to impaired Cr biosynthesis, we also measured kidney production of guanidinoacetic acid (GAA). No difference was observed between CrT ^–/y^ (9.76 ± 0.71 nmol/mg of protein) and CrT ^+/y^ mice (10.70 ± 0.63 nmol/mg of protein; t test, p = 0.359).

**Table 1.** Depletion of Cr levels in CrT ^–/y^ mutant mice. Cr levels (mean ± SEM) in CrT ^–/y^ and CrT ^+/y^ animals (n = 4 per tissue for both groups). Cr levels have been measured by GC/MS. A reduction of Cr content was evident in the brain, muscle, heart and kidney tissue of mutant animals (Two Way ANOVA on ranks, post hoc Holm-Sidak method). * p < 0.05; ** p < 0.01; *** p < 0.001.

Tissue (nmol/mg protein)	CrT ^–/y^	CrT ^+/^
Cerebral cortex	13.61 ± 1.06**	76.36 ± 3.16
Hippocampus	14.14 ± 1.52***	83.69 ± 4.37
Muscle	111.57 ± 21.27**	310.20 ± 31.59
Heart	1.19 ± 0.27***	89.92 ± 5.15
Kidney	1.59 ± 0.13*	9.60 ± 0.65

### Reduced body weight growth in CrT ^–/y^ mice at two months of age

**Figure 2.**
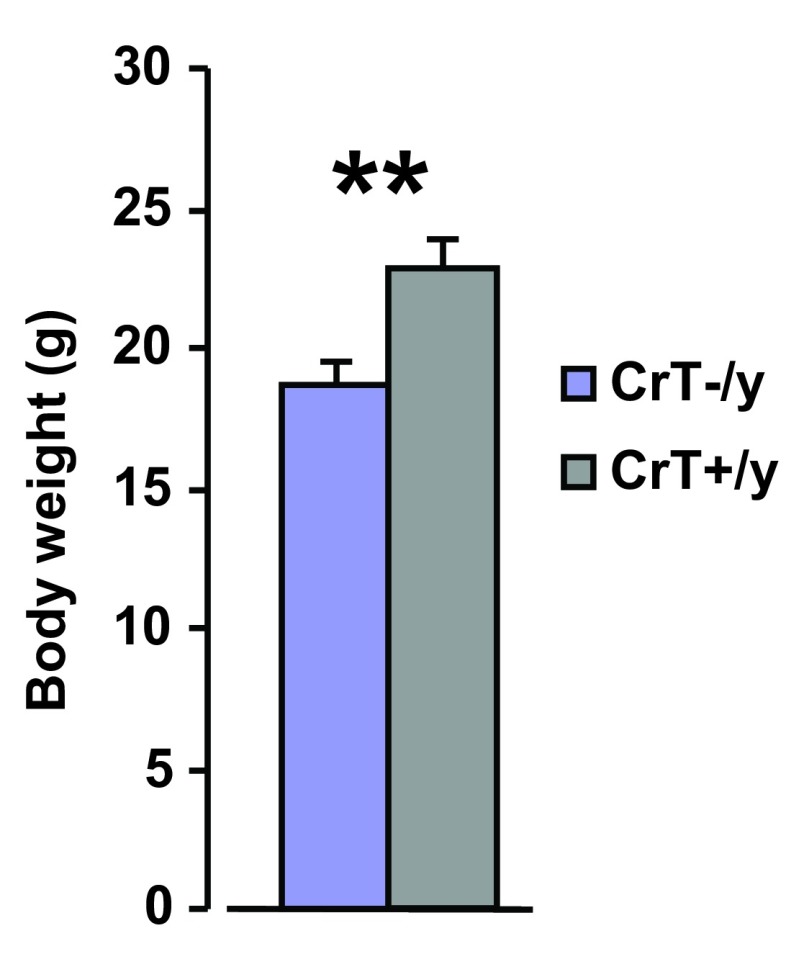
Body weight is lower in CrT ^–/y^ animals at two months of age. At P60 the weight of CrT ^–/y^ mice was significantly reduced compared to CrT ^+/y^ animals (CrT ^–/y^: 18.75 ± 0.78 g, CrT ^+/y^: 22.77 ± 0.90 g; t test, p < 0.01). *, statistical significance. Error bars, s.e.m.

The general appearance of CrT ^–/y^ mice was normal and no particular problems of breeding were observed. To evaluate the effects of CrT deletion on body weight, the mice with targeted disruption of CrT gene were weighed at P60, and compared with WT littermates. CrT ^−/y^ animals (n = 9) showed a significantly reduced body weight compared to CrT ^+/y^ animals (n = 9; t test, p < 0.01; Figure 2).

### Normal behavior of CrT ^–/y^ mice in the open field arena

**Figure 3.**
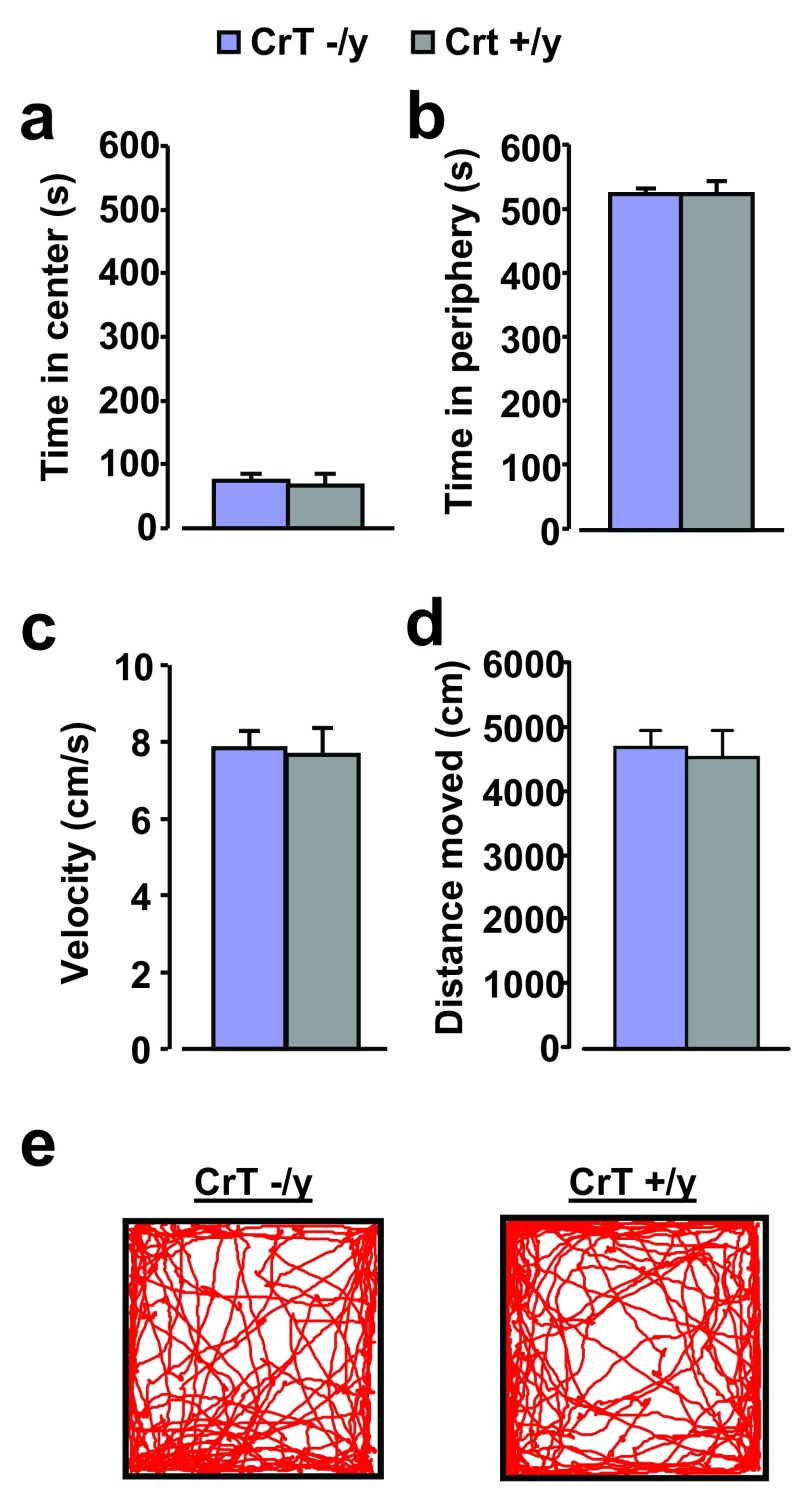
Normal behavior of CrT mutant mice in the open field arena. ( **a**, **b**) CrT ^–/y^ (n = 9) and CrT ^+/y^ mice (n = 9) spent a comparable amount of time in the center (CrT ^–/y^: 75.16 ± 10.82 s, CrT ^+/y^: 67.60 ± 18.11 s; **a**) and in the peripheral region (CrT ^–/y^: 524.41 ± 10.87 s, CrT ^+/y^: 526.52 ± 18.45 s; **b**) of the open field arena. A Two-Way ANOVA analysis shows no significant effect of genotype for both comparisons (p = 0.725 and p = 0.922, respectively). ( **c**) Walking speed of animals during the exploration of open field arena. We found no significant difference (CrT ^–/y^: 7.85 ± 0.43 cm/s, CrT ^+/y^: 7.65 ± 0.71 cm/s; t test, p = 0.807). ( **d**) The total distance moved in the open field arena did not differ between CrT mutants (4706.34 ± 258.75 cm) and WT animals (4535.28 ± 427.11 cm; t test, p = 0.736). ( **e**) Representative examples of movement path during the open field session for a CrT ^–/y^ (left) and a CrT ^+/y^ mouse (right). Error bars, s.e.m.

We first analyzed the general motor activity and anxiety-related behavior of CrT ^−/y^ (n = 9) and CrT ^+/y^ mice (n = 9) in the open field arena. Even though both groups of animals tended to avoid the center of the arena, remaining in the peripheral region for a significantly longer duration (Two Way ANOVA, post hoc Holm-Sidak method), the time spent by CrT ^−/y^ mutant mice in both the central and peripheral portion of the apparatus was not different from that recorded for WT animals (Two Way ANOVA, post hoc Holm-Sidak method, p = 0.725 and p = 0.922 respectively; Figure 3a, b, e). No difference between CrT ^−/y^ and CrT ^+/y^ animals was present even in motion speed and total distance moved (t test, p = 0.807 and p = 0.736 respectively; Figure 3c, d).

### CrT ^–/y^ mice display declarative memory deficits in the object recognition test

**Figure 4.**
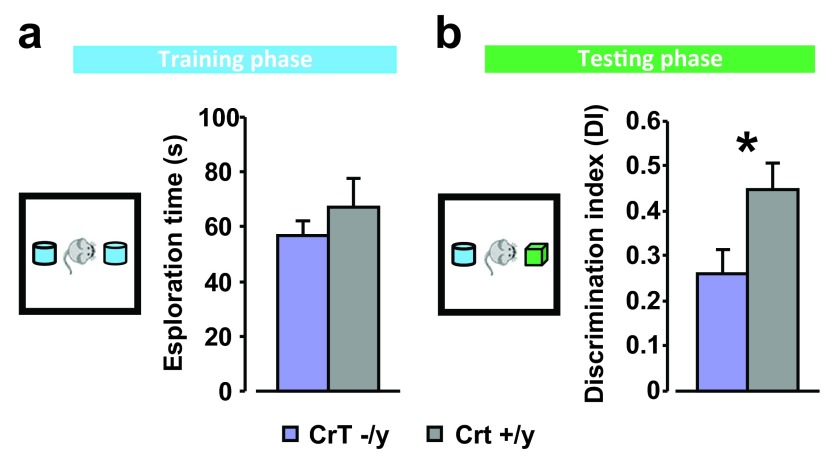
CrT deletion leads to cognitive deficits in object recognition memory. ( **a**) On the left, a schematic representation of the sample condition in object recognition task. Histograms depict the performance of CrT ^–/y^ and CrT ^+y^ during the sample phase: no difference in the total exploration time of objects was detected between the experimental groups (CrT ^–/y^: n = 8, exploration time = 56.91 ± 5.40 s; CrT ^+/y^ : n = 6, exploration time = 67.20 ± 10.23 s; t test, p = 0.358). ( **b**) On the left, a schematic diagram of the test condition. Histograms display object discrimination indexes of CrT ^–/y^ and CrT ^+y^ during the testing phase: a significantly lower discrimination index was found in CrT ^–/y^ mice (0.261 ± 0.053) compared to CrT ^+y^ animals (0.448 ± 0.059; t test, p < 0.05). *, statistical significance. Error bars, s.e.m.

We assessed declarative memory abilities in the object recognition test (ORT) evaluating animal ability to discriminate a new versus a familiar object. During the sample phase ( Figure 4a), all experimental groups equally explored the objects, with a total exploration time of mutant mice (n = 8) very close to that recorded for the control group (n = 6; t test, p = 0.358). After a delay of 24h, the testing phase revealed that while CrT ^+/y^ mice displayed a clear preference toward the novel object spending a significantly longer time exploring it, an impaired performance was found in CrT ^–/y^ animals, which exhibited a significantly lower discrimination index than control animals (t test, p < 0.05, Figure 4b).

### Impaired spatial working memory in CrT ^–/y^ mice

**Figure 5.**
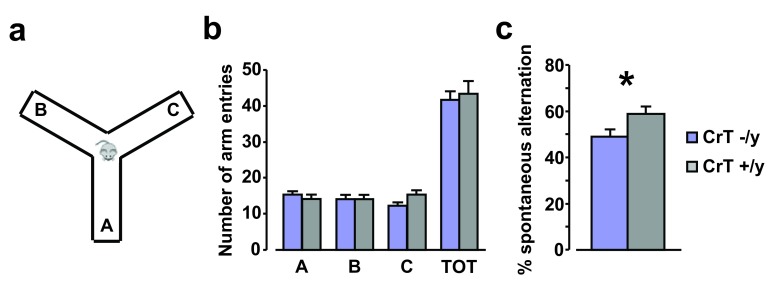
Impairment of Y-maze spontaneous alternation rate in CrT ^–/y^ mice. ( **a**) Schematic diagram of the Y maze apparatus. ( **b**) Histograms depict the mean number of entries in the single arms of the maze (A, B, C) and the total number of arm entries for the different experimental groups: animals of both groups equally explored all the three arms of the maze and general exploratory behavior of CrT ^–/y^ animals (n = 9; A: 15.22 ± 1.12, B: 14.22 ± 1.08, C: 12.22 ± 1.05, TOT: 41.67 ± 2.41) was totally comparable to that exhibited by WT littermates (n = 9; A: 14.00 ± 1.26, B: 14.11 ± 1.29, C: 15.22 ± 1.27, TOT: 43.33 ± 3.58; Two-Way ANOVA, post hoc Holm-Sidak method, p = 0.640, p = 0.966, p = 0.252, p = 0.523 respectively). ( **c**) Alternation rate in the Y maze was significantly lower in CrT ^–/y^ mice (49.24 ± 3.20%) compared to that recorded for CrT ^+/y^ littermates (58.91 ± 2.99%; t test, p < 0.05). *, statistical significance. Error bars, s.e.m.

To evaluate whether CrT deletion may affect spatial working memory, we used the analysis of spontaneous alternation in the Y maze ( Figure 5a). Animals of both groups equally explored all the three arms of the maze. Indeed, no effect of genotype was detected for either the number of entries in the single arms of the maze (designated A, B, C) or the total number of arm entries, indicating that the exploratory disposition of mutant animals (n = 9) was not altered compared to WT littermates (n = 9; Two-Way ANOVA, post hoc Holm-Sidak method, p = 0.640, p = 0.966, p = 0.252, p = 0.523 respectively, Figure 5b). In contrast, CrT ^−/y^ mice showed a significantly smaller rate of spontaneous alternation with respect to WT controls (t test, p < 0.05, Figure 5c).

### CrT deletion impairs spatial learning and memory in mutant mice

**Figure 6.**
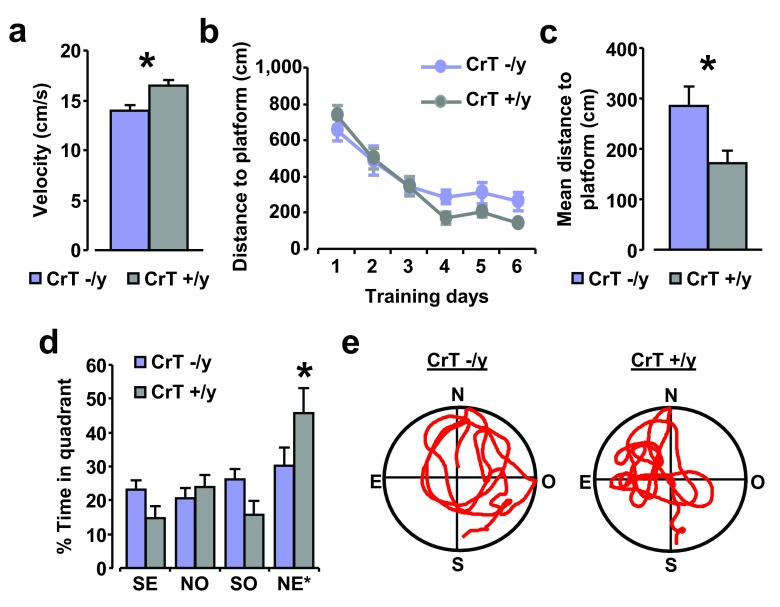
CrT deletion impairs spatial learning and memory in mutant mice. ( **a**) Mean swimming speed measured all along the training phase for CrT ^–/y^ and CrT ^+/y^ animals: mutant mice (14.00 ± 0.53 cm/s) resulted to be slower swimmers with respect to control littermates (16.44 ± 0.60 cm/s; t test, p < 0.05). ( **b**, **c**) Learning curves for CrT ^–/y^ (n = 9; blue) and CrT ^+/y^ mice (n = 5; grey) during the training phase. The histogram shows the mean swimming path covered to locate the submerged platform on the last three day of training for the two groups. A t-test analysis showed a statistical difference between CrT ^–/y^ (285.24 ± 37.53 cm) and CrT ^+/y^ animals (171.58 ± 23.80 cm; p < 0.05). ( **d**) Probe trial. A Two-Way RM ANOVA followed by Holm-Sidak multiple comparison revealed that while CrT ^+/y^ spent significantly more time in the NE quadrant than in the other ones, CrT ^–/y^ did not show any preference for the target quadrant. In addition, the percentage of time spent in the target quadrant was shorter in CrT ^–/y^ mice (30.31 ± 5.33%) than in the other group (45.73 ± 7.35%). ( **e**) Representative examples of swimming path during the probe session for a CrT ^–/y^ (left) and a CrT ^+/y^ mouse (right). *, statistical significance. Error bars, s.e.m.

We further assessed spatial memory abilities in the Morris water maze (MWM) task, a cognitive paradigm which allows testing both spatial learning and memory. Since a main effect of genotype was found on mean swimming speed recorded all along the training phase (t test, p < 0.05; Figure 6a), we analyzed path length, which is a quantity independent of swimming velocity. We found that the mean distance covered to locate the submerged platform on the last three days of training was longer in CrT ^–/y^ mice (n = 9) compared to CrT ^+/y^ littermates (n = 5; t test, p < 0.05; Figure 6b, c). To measure the strength of spatial learning and to discriminate between spatial and non-spatial memory strategies we performed a probe trial in which the hidden platform was removed and the amount of time spent in the former region of the platform was measured. The probe test confirmed the spatial memory impairment of CrT ^–/y^ mice: CrT ^+/y^ animals spent significantly longer time in the quadrant where the platform was located during the previous learning days (NE*; Two-Way RM ANOVA, post hoc Holm-Sidak method, p < 0.05 for all comparisons); in contrast, CrT ^–/y^ mice showed no preference for the target quadrant, indicating that they did not remember the location of the hidden platform (Two-Way RM ANOVA, post hoc Holm-Sidak method; Figure 6d). A statistically significant effect of genotype was detected in the time spent exploring the target quadrant (Two-Way RM ANOVA, post hoc Holm-Sidak method, p < 0.05; Figure 6d).

### Cr depletion reduces spontaneous locomotor activity in CrT ^−/y^ mice

**Figure 7.**
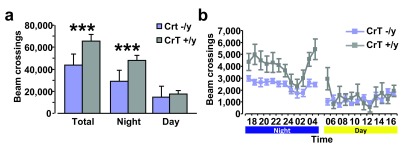
Locomotor activity in CrT ^–/y^ mutant mice and the wild-type parental strain. ( **a**) Total horizontal distance travelled throughout 24h (left), and over the dark (middle) or light phase (right). CrT ^–/y^ mice had a significant decrease in motor activity in comparison to control animals during the whole period of testing (CrT ^–/y^: 43,594.22 ± 2,639.39 beam crossings, CrT ^+/y^: 65,587.63 ± 5,831.19 beam crossings) and the night phase (CrT ^–/y^: 29,109.67 ± 1,695.35 beam crossings, CrT ^+/y^: 48,094.13 ± 4,843.56 beam crossings; Two-Way ANOVA, post hoc Holm-Sidak method, p < 0.001 for both comparisons), while the motor behavior of the two groups was similar in the day-time (CrT ^–/y^: 14,484.56 ± 1,458.08 beam crossings, CrT ^+/y^: 17,493.50 ± 2,957.57 beam crossings; p = 0.535). ( **b**) Time course of horizontal activity of CrT ^–/y^ (blue) and CrT ^+/y^ (grey) animals during 24h. Data are plotted as total number of beam crossings ± SEM in each time block of 60 min. Dark and light phases are indicated. *, statistical significance. Error bars, s.e.m.

To investigate the presence of movement impairments in CrT ^−/y^ mice in a non-aversive environment, we investigated home-cage-locomotor activity. We found that CrT ^–/y^ mice (n = 9) are significantly less active than the CrT ^+/y^ group (n = 8, Two-Way ANOVA, post hoc Holm-Sidak method, p < 0.001). More specifically, CrT ^−/y^ mice showed decreased horizontal activity during the night period (Two-Way ANOVA, post hoc Holm-Sidak method, p < 0.001), while no effect of genotype was observed for exploration during daytime (p = 0.535; Figure 7a, b).

Data for neurochemical and behavioral assessment in a mouse model of creatine transporter deficiency Detailed descriptions of each dataset can be found in the text file provided.

## Discussion

We have generated a new murine model of human CrT deficiency carrying a loss of function deletion of 5–7 exons in the murine orthologous of *Slc68a* gene. Given that most disease-underlying mutations in human CCDS1 lead to loss of CrT function ( van de Kamp *et al.*, 2014), our model has a good degree of construct validity. Beyond the genetic deletion, neurochemical abnormalities found in CrT ^−/y^ mice, reproducing the reduced levels of Cr that characterize the brain of CCDS1 patients ( van de Kamp *et al.*, 2012), are also helpful to confirm the successful disruption of CrT gene and the construct robustness of this model. Importantly, Cr deficiency is apparent in both the cerebral cortex and hippocampus, i.e., two brain regions crucially involved in the patient cognitive defects. These results seem to support the hypothesis that, despite AGAT and GAMT expression ( Carducci *et al.*, 2012; Schmidt *et al.*, 2004; Tachikawa *et al.*, 2004), in CrT deficiency conditions endogenous synthesis does not compensate for the loss of Cr uptake in the mouse ( Skelton *et al.*, 2011) as in the human brain ( Cecil *et al.*, 2001). In contrast to the preservation of Cr levels in skeletal muscle of CCDS1 patients ( deGrauw *et al.*, 2003), we observed that mutant mice exhibit Cr reductions in muscle and other peripheral tissues. This observation is in agreement with data showing that skeletal muscle tissue from a different CrT knockout mouse displayed a dramatic reduction of Cr levels ( Russell *et al.*, 2014; Skelton *et al.*, 2011).

Our behavioral investigation highlighted that CrT ^−/y^ mice carrying a different deletion than previously reported ( Skelton *et al.*, 2011) exhibit a broad spectrum of phenotypes establishing the validity of this model and corroborating its utility in translational studies. Mutant mice, indeed, show cognitive impairments in a battery of learning and memory tests aimed at assessing both explicit and implicit memories such as object-recognition task, Y maze and Morris water maze. The memory deficiency assessed across a variety of behavioral tasks indicates that CrT ^−/y^ animals have a general cognitive impairment, which is a key clinical feature in CCDS1 patients.

While the motor development is only mildly delayed in CCDS1 patients ( van de Kamp *et al.*, 2013) and myopathic symptoms have been rarely described ( Anselm *et al.*, 2006; van de Kamp *et al.*, 2013), mostly as late onset deficits ( deGrauw *et al.*, 2002; Hahn *et al.*, 2002; Kleefstra *et al.*, 2005), we found that reduced muscle levels of Cr measured in mutant animals were accompanied by alterations of motor behavior. CrT ^−/y^ mice, indeed, showed significantly decreased home-cage-locomotor activity (particularly evident during the night period) and they were slower swimmers than CrT ^+/y^ mice. In contrast, we found that vulnerability to stress and anxiety responses are not sensitive to CrT deletion. Future studies using conditional mouse models with a disruption of CrT allele only in the brain tissue will be useful to dissect the role of peripheral Cr in the development of cognitive deficits. It has been reported that a CrT deletion exclusively restricted to forebrain excitatory neurons during late postnatal development induces selective learning and memory deficits without affecting motor behavior ( Kurosawa *et al.*, 2012).

Because of the importance of Cr in normal retinal function and development ( Acosta *et al.*, 2005), it has been suggested that an alteration of visual capabilities might play a role in the cognitive deficits displayed by CrT ^−/y^ animals. We reported that during the ORT sample phase all experimental groups equally explored and observed the objects, with the total exploration time of mutant mice very close to that recorded for the control group, suggesting that the visual system is not impaired in CrT ^–/y^ animals. In addition, to avoid possible confounding effects due to reduced visual acuity, the tank used in the Morris water maze task was surrounded by a set of extra-maze cues in a visual discrimination range detectable even by partially-sighted animals.

In conclusion, this CrT ^−/y^ murine model will provide a new tool for improving preclinical evaluation of potential CCDS1 intervention treatments. The results confirm previous data suggesting that CCDS1 can be well modeled in mice ( Kurosawa *et al.*, 2012; Skelton *et al.*, 2011). Null mice display an impairment of motor behavior rarely present in human patients; however, the use of conditional mice will avoid this problem. Since CCDS1 is still an untreatable pathology, there is a compelling need for developing effective therapeutic strategies. The availability of murine models that reliably reproduce the human condition will fuel and support the research in this field. To assess the reproducibility and the predictive validity of promising treatments for CCDS1 as well as for other disorders, the validation of findings in more than one animal model is strongly desirable prior to launching later-stage translational or clinical projects ( Katz *et al.*, 2012). Since another invalidating hallmark of CCDS1 is the frequent occurrence of seizures, additional studies in CrT ^−/y^ mice analyzing the behavioral response to kainic-acid injection will be required to provide useful information about seizure susceptibility in this model.

## Data availability

*F1000Research*: Dataset 1. Data for neurochemical and behavioral assessment in a mouse model of creatine deficiency, 10.5256/f1000research.5369.d36153 ( Baroncelli *et al.*, 2014).
