# Supplementary material for: A novel mouse model of creatine transporter deficiency
Source: F1000Res. 2014 Sep 29;3:228. [Version 1] doi: 10.12688/f1000research.5369.1 (PMC4243761; doi:10.12688/f1000research.5369.1)
Supplement: Peer review history

# Peer review: Referee response for version 1

**DOI:** 10.5256/f1000research.5732.r6559

**Published:** 2014-11-11

**Authors:** Andreas Schulze

**Affiliations:** 1 Division of Clinical and Metabolic Genetics, Department of Pediatrics, University of Toronto, Toronto, Canada

The research group generated a new ubiquitous CrT knockout as mouse model for creatine transporter deficiency with a large 3 exon deletion in the Slc6a8 gene. Biochemical phenotyping revealed creatine deficiency in brain, muscle, heart, and kidneys and behavioral testing revealed a phenotypic similarities with CrT patients. Therefore the new mouse model appears to be a valid tool to study creatine transporter deficiency.

What needs some more elaboration is the discrepancy in findings compared to the mouse model described by Skelton *et al. (*2011). Considering the similarities of the knock-outs, both have a large deletion of three exons, on would expect similar findings. But in the knock-out presented here there is more cognitive impairment, i.e. novel object recognition was abnormal while it was normal in the Skelton paper, and this is despite of the fact that the brain creatine deficiency reported here appears to be less pronounced than in the mouse model of Skelton *et al*.

Before indexing the authors should provide information on whether the mouse chow contained creatine (some mouse chow contains fish meal and the latter contains creatine). Also it would be important to know the creatine concentration in plasma. The creatine concentration in mutants is expected to be higher than in wild types. I wonder whether blood contamination has contributed to unexpected high brain creatine concentration in mutants. Why did the group not consider whole-body perfusion prior to organ removal? Did the authors measure creatine/creatinine ratios in urine? And why not providing the information on guanidinoacetate in organs and body fluids as well?

I have read this submission. I believe that I have an appropriate level of expertise to confirm that it is of an acceptable scientific standard, however I have significant reservations, as outlined above.

# Peer review: Referee response for version 1

**DOI:** 10.5256/f1000research.5732.r6560

**Published:** 2014-11-05

**Authors:** Luis M. Valor

**Affiliations:** 1 Instituto de Neurociencias de Alicante (Universidad Miguel Hernández - Consejo Superior de Investigaciones Científicas), Alicante, Spain

In “A novel mouse of creatine transporter deficiency” the authors describe the phenotype of a new knockout mouse for *Slc6a8* gene which is associated with a depletion in the levels of creatine in diverse organs. This phenotype is reminiscent of the CCDS1 symptomatology, therefore the main output of the present report is an increase in the number of available murine models for this disorder as claimed in the article.

The paper is well written and the work is well presented, with no major concerns regarding the data as shown. Nonetheless, I miss a more complete behavioural analysis. In some cases this is not crucial because assessment of particular tasks is expected to support current findings (absence of anxiety in the open field or impaired spatial working memory in the Y-maze) although with the strength of using more dedicated paradigms (elevated plus maze or T-maze based on a rewarding system, respectively). However, it is more relevant in the case of motor and neuromuscular deficits to enhance the conclusions obtained from spontaneous activity measurements, and other approaches (accelerating rotarod, grip strength, vertical pole, etc. to put some examples) may be more informative.

I have read this submission. I believe that I have an appropriate level of expertise to confirm that it is of an acceptable scientific standard.

# Peer review: Referee response for version 1

**DOI:** 10.5256/f1000research.5732.r6247

**Published:** 2014-10-02

**Authors:** Benedetto Sacchetti

**Affiliations:** 1 Department of Neuroscience, University of Turin, Turin, Italy

In the present paper the authors describe a new murine model of CCDS1 obtained by ubiquitous deletion of 5-7 exons in the *Slc6a8 *gene. The experiments in general are well controlled and the results could be of interest for a general audience. However, I think there are two related points that should be added or clarified before the paper can be considered for publication.

The authors reported that Cr depletion altered spontaneous locomotor behavior (Fig 7) and reduced muscle levels. How can this data fit with the normal behavior (namely, the motion speed and the total distance moved) displayed by mutant animals in the open field test?

On the other hands, can the authors exclude that the aforementioned reduced motor activity may have interfere with learning and memory trials? The authors should at least discuss this possibility.

I have read this submission. I believe that I have an appropriate level of expertise to confirm that it is of an acceptable scientific standard, however I have significant reservations, as outlined above.

# Response

**Published:** 2014-11-06

**Authors:** Laura Baroncelli

**Affiliations:** CNR Institute of Neuroscience, Italy, Italy

- We agree that the difference between the altered spontaneous locomotor behavior of mutant animals in the home cage and the normal exploratory disposition in the open field arena can be a little bit surprising. However, we feel that the lack of a genotype effect for the latter measure may be due to the aversive nature of the open field arena, which may affect the explorative behavior of both wild-type and mutant mice, thus masking the difference in motor activity between the two groups.

- We think that our data allow to exclude the possibility that an impaired motor activity can interfere with the presented results of learning and memory test. We found that, during the ORT sample phase, the total exploration time of objects was equal for mutant and control mice (Figure 4a), and animals of both groups equally explored the Y maze in terms of both the number of entries in the single arms of the maze and the total number of arm entries (Figure 5b). These results strongly suggest that animals’ level of activity does not affect their cognitive performance. As for the Morris water maze during the training phase we analyzed the path length covered to locate the submerged platform just to avoid the confounding effects of the reduced swimming speed observed in mutant mice that should not affect instead the performance in the probe trial.
